# Metabolic and Biochemical Stressors in Diabetic Cardiomyopathy

**DOI:** 10.3389/fcvm.2017.00031

**Published:** 2017-05-31

**Authors:** Vasundhara Kain, Ganesh V. Halade

**Affiliations:** ^1^Division of Cardiovascular Disease, Department of Medicine, University of Alabama at Birmingham, Birmingham, AL, United States

**Keywords:** cardiomyopathy, diabetes, fatty acids, inflammation, hypertension, cardiac remodeling

## Abstract

Diabetic cardiomyopathy (DCM) or diabetes-induced cardiac dysfunction is a direct consequence of uncontrolled metabolic syndrome and is widespread in US population and worldwide. Despite of the heterogeneous and distinct features of DCM, the clinical relevance of DCM is now becoming established. DCM progresses to pathological cardiac remodeling with the higher risk of heart attack and subsequent heart failure in diabetic patients. In this review, we emphasize lipid substrate quality and the phenotypic, metabolic, and biochemical stressors of DCM in the rodent and human pathophysiology. We discuss lipoxygenase signaling in the inflammatory pathway with multiple contributing and confounding factors leading to DCM. Additionally, emerging biochemical pathways are emphasized to make progress toward therapeutic advancement to treat DCM.

## Introduction

Cardiovascular disease (CVD) is the primary cause of death including substantial people suffering from obesity and type 2 diabetes. With an increased population of patients displaying metabolic syndrome, there are 17.3 million deaths per year, and this number is expected to increase to more than 23.6 million by 2030 (1, 2). Diabetes and obesity are primary metabolic triggers associated with imbalanced energy [fatty acids (FAs)] intake, which is an inherent part of the modern lifestyle. Obese people are often prone to or diagnosed with insulin resistance, pre-diabetes, impaired glucose tolerance, or type 2 diabetes. According to current statistics, nearly 29 million individuals in US have diabetes, and one in three adults has the pre-diabetic condition (3) and heart disease, and stroke is leading cause of disability, morbidity, and death among people with accelerated or uncontrolled type 2 diabetes. CVD is associated with ~65% of deaths related to diabetes (4) and has an adverse effect on left ventricle size, geometry, and function leading to diabetic cardiomyopathy (DCM).

Diabetic cardiomyopathy is a complex pathological as well as adaptive condition characterized by dysfunctional effects on the left ventricle and is developed by a combination of several metabolic disorders including prediabetes, hyperglycemia, insulin resistance (in type 2 diabetes), hypertension, and obesity (5). There are multiple factors and mechanisms including aging that aggravate the pathology of DCM. However, in the current context of cardiomyopathy, DCM is defined by presence of abnormal myocardial diastolic or systolic function in the presence of diabetes without known hypertension or coronary artery disease (6). It has been categorized and presented in different manners as it includes features of left ventricular (LV) hypertrophy, myocardial fibrosis, and myocardial energy dysregulation with differential degrees of myocardial biochemical, mechanical, or structural dysfunction. Due to its multifactorial origin and distinct pathophysiology, there are some controversies regarding the existence or non-existence of DCM. Clinically, DCM lacks classical features of a cardiomyopathy such as ventricular dilation and meaningful systolic dysfunction. From metabolism perspective, DCM is a combination of molecular myocardial abnormalities that lead to the development of myocardial dysfunction with co-existence of additional stressors such as obesity, hypertension, and coronary artery disease. In this review, we focus on the etiology that aggravates DCM in clinical and pre-clinical settings. We discuss pathophysiological and metabolic stressors that are prime contributors to DCM. In order to focus on the development and advancement of therapeutic targets in the DCM field, we asked three questions: (1) Do we need to treat the original conditions or metabolic abnormalities of DCM in order to reduce or prevent DCM and heart failure? (2) Do we need to develop a pharmacological lifestyle or targeted aggressive metabolomics approach to identify the biomarker signatures to treat DCM? (3) Does a combination of (1) and (2) allow adequate control of the original metabolic abnormality with focused treatment of DCM, particularly in an aging population?

## Metabolic Stressors for the Development of DCM

In order to understand the pathophysiology of DCM and its etiology related to metabolic remodeling in the heart, we have highlighted the major confounding causes below and summarized experimental rodent models used to understand the dysregulation in the latter (Table 1), acknowledging that the scope of this review does not allow us to discuss them all.

**Table 1:** **Metabolic dysregulations in diabetic cardiomyopathy**.

Phenotypic parameters	STZ	OVE26	BB	NOD	KKA^y^	Alloxan	Akita	ob/ob	db/db	ZDF
Obesity	−	−	−	−	+	−	+	+	+	+
Diabetes	++	+	+	+	−	+	+	+	+	+
Hypertension	−	−	−	−	+	−	−	+/−	−	−

*+, positive for metabolic pathology; ++, highly positive for metabolic pathology; −, negative for metabolic pathology*.

*STZ, streptozotocin; OVE26, beta-cell overexpression of calmodulin; BB, Biobreeding rat; NOD, non-obese diabetic; KKA^y^, yellow KK obese; ob/ob, leptin-deficient obese mice; db/db, type 2 diabetic Lepr deficient; ZF/ZDF, Zucker fatty/Zucker diabetic fatty rats*.

### Obesity

**Figure 1:**
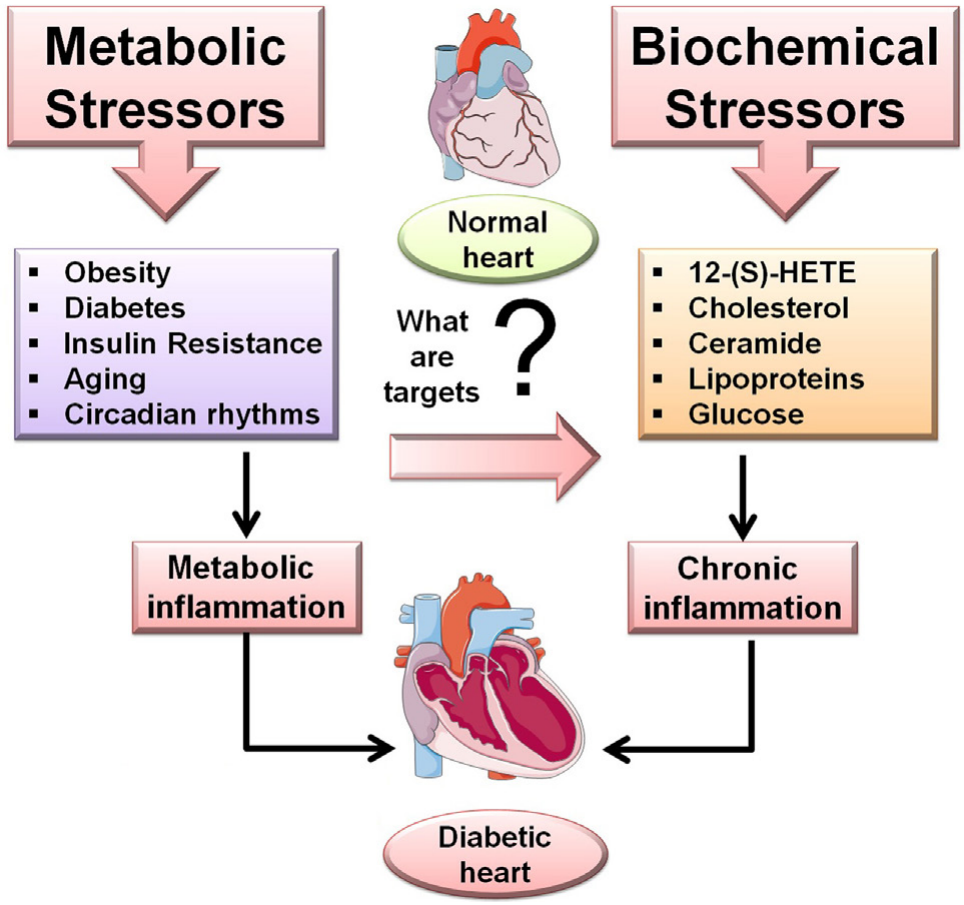
**Overview of the metabolic and biochemical stressors in diabetic cardiomyopathy (DCM)**. Metabolic stressor such as obesity, diabetes, insulin resistance, aging, and circadian rhythms while the biochemical stressors 12-(S)-HETE, cholesterol, ceramide, lipoproteins, glucose trigger inflammation-mediated DCM.

Obesity is characterized by a low-grade chronic inflammation phenotype with an excessive amount of body fat leading to heart disease, diabetes, and high blood pressure. More than 34.9%, almost 78.6 million of US adults are obese (7). Obese and overweight individuals are prone to insulin resistance and diabetes, accompanied frequently by LV eccentric or concentric hypertrophy (8). Obesity is characterized on the basis of body mass index (BMI) and has been proved to be associated with ventricular hypertrophy (9, 10). The association of obesity with systolic dysfunction and with the prevalence of diabetes makes obesity a confounding factor of DCM. The predicted body weight along with the excess fat mass accounts for a rise in LV stroke volume and stroke works due to accelerated heart rate. The Framingham study suggested that a BMI >30 kg/m^2^ is positively correlated with increased LV wall thickness, LV internal dimension in diastole, and LV mass (11). A number of studies have shown that visceral adipose tissue has higher levels of pro-inflammatory cytokines when associated with LV diastolic dysfunction (12). Studies performed on the isolated hearts of genetic animal models of obesity, such as Zucker fatty (ZF) rats or leptin-deficient obese mice, showed a depression in cardiac function (13, 14). However, a few studies also reported the normal function of the heart in obese rodent models (15, 16). As type of fat, duration, and dose consumed impacts LV structure, function, and healing in the myocardial infarction (MI)-induced model, the translational prospects of fat intake and intervention studies should be used with caution. This is exemplified by studies from Brainard and colleagues suggesting that a high-fat diet in the form of lard or milk before and after MI is insufficient to induce cardiac dysfunction, despite adiposity and impaired glucose disposal (17). The study showed that the signs of cardiac dysfunction are not visible in commonly used mouse model [type 2 diabetic Lepr-deficient (db/db) mice or streptozotocin (STZ)-treated wild-type mice] when subjected to pressure overload. But the db/db model showed depressed cardiac function when subjected to ischemia–reperfusion injury in comparison with non-diabetic mice (17). By contrast, when the obesity is superimposed on aging that is the trigger to develop non-resolving inflammation post-MI. Recent study by Lopez et al. highlighted that supplementation of n-6 FAs in aged mice resulted in higher levels of 12-*S*-hydroxyeicosatetraenoic acids (HETE) leading to an acute inflammatory response, further delaying healing in MI (18). These results are similar to the findings that showed higher levels of 12-HETE in individuals with stable angina (19). Studies of aging coupled with obesity have shown that the n-6 enriched fat diet creates lipid metabolites that are pro-inflammatory in nature (Figure 1). Aging mice fed a diet enriched in linoleic acid showed increased neutrophils in the gut and development of dysbiosis (20). Thus, these metabolites and their action in disease pathology such as pressure overload, post-MI healing, and overall cardiac remodeling will provide novel tools for drug discovery and target identification.

### Metabolic Syndrome, Diabetes, and Insulin Resistance

Type 2 diabetes is defined as a metabolic syndrome with a combination of insulin resistance and defective insulin secretion by pancreatic β-cells. The Diabetes Control and Complications Trial/Epidemiology of Diabetes Interventions and Complications (DCCT/EDIC) study was important in showing the role of hyperglycemia in micro- and macro-vascular diabetes-mediated complications (21, 22). Impaired insulin sensitivity results from obesity and physical inactivity or genetic susceptibility (23, 24). Currently, 39 million Americans are diabetic, predisposing them to cardiovascular risk. Clinical data have demonstrated that diabetes is an independent risk factor for cardiovascular complications. In both men and women, diabetes has been observed to be one of the primary causes of DCM and subsequent heart failure over the last three decades (5, 25). The development of diabetes with LV dysfunction was clinically evident 20 years earlier (26). Many clinical studies have shown that more than 60% of diabetic patients compared with well controlled subjects are diagnosed with early and mild ventricular diastolic dysfunction (27, 28). Studies have confirmed that DCM is not an unusual condition but caused by low-grade non-resolving inflammation, uncontrolled hyperglycemia (glucotoxicity), or hyperlipidemia (lipotoxicity). These factors mainly contribute to metabolic syndrome, leading to cardiac dysfunction and increasing the risk of MI or recurrent MI (29). Glucotoxicity refers to uncontrolled hyperglycemia and subsequent metabolic changes triggered by excess sugar or carbohydrate (30). Lipotoxicity is more complex and diverse because of the essential nature of FAs, but excessive intake in aging may lead to bone marrow adiposity and non-resolving inflammation post-MI (31–35). The consequences of glucotoxicity or lipotoxicity considering the metabolic health and physical inactivity of a diverse human population are key challenges in the prevention and treatment of DCM (36). The Framingham Heart Study showed that the frequency of heart failure is doubled in diabetic men and quintupled in diabetic women in comparison with age-matched control subjects. Clinical studies have shown that diabetic subjects have a high risk of cardiac failure accompanied with systolic dysfunction and LV hypertrophy (37, 38). Number of surveys and studies have shown a higher prevalence of diastolic dysfunction in diabetic patients (39, 40). Rodent and clinical studies in diabetes setting have shown functional and structural alterations in myocardium tissue. Studies performed in type 1 diabetic model, i.e., STZ-induced diabetic mouse, and rodents with type 2 diabetes, i.e., ZF rats or db/db mice, have shown diastolic dysfunction (Table 2) (13, 41). In fat and fructose-feeding studies, serum uric acid levels led to an increase in cardiac tissue xanthine oxidase activity, which was temporally related to an increase in body weight, fat mass, and insulin resistance without changes in blood pressure, when these mice were subjected to excess fat (46%) and fructose (17.5%) for 16 weeks. The high-fat and fructose diet led to cardiomyocyte hypertrophy, oxidative stress, interstitial fibrosis with impaired diastolic relaxation, and macrophage polarization toward a pro-inflammatory phenotype (42). Recent studies in many models have shown increases in levels of the 12/15-lipoxygenase (LOX) pro-inflammatory intermediate metabolite 12-HETE (43, 44). Insulin resistance is the prime risk factor to initiate the defects in insulin signaling pathways and glucose transport to cells. The resistance of the body toward insulin results in increased production of insulin in the pancreas leading to hyperinsulinemia (45). An association between insulin resistance and heart failure was noted a century ago and later traced to metabolic alteration (46).

**Table 2:** **Biochemical dysregulations in diabetic cardiomyopathy**.

Functional and metabolic outcomes	Type 1 diabetes					Type 2 diabetes			
	STZ	OVE26	BB	NOD	Alloxan	Akita	ob/ob	db/db	ZDF
Inflammation	↑	↑	↑	↑	↑	↑	↑	↑	↑
Cardiac size	=	=	=	↑	=	↓	↑	↑	↑
Cardiac function	↓	↓	↓	↓	↓	↓	↑/↓	↓	↓
Cardiac efficiency	↓	=	=	↓	↓	=	↓	↓	=
Mitochondrial energetics	↓	↓	↓	=	↓	↓	↓	↓	=
Lipid storage	↑	=	=	↑	=	=	↑	↑	↑
Fatty acid oxidation	↑	=	=	↑	=	↑	↑	↑	↑
Glucose oxidation	↓	=	=	=	=	↓	↓	↓	↓

*Up and down arrows indicate increase and decrease, respectively; =, indicates no change*.

*OVE26, beta-cell overexpression of calmodulin; NOD, non-obese diabetic; STZ, streptozotocin; ZF/ZDF, Zucker fatty/Zucker diabetic fatty rats*.

Studies have shown that insulin resistance is a common factor in patients with non-ischemic cardiomyopathy compared with control populations, which exclude pre-existing diabetes patients (47). A study of 1,187 Swedish people showed that heart failure could be anticipated in the patients with insulin resistance without any prior heart failure history, excluding all other factors (48). Lack of insulin response can lead to inactivation of cellular pathways such as AKT inactivation, reduced nitric oxide production, and increased apoptosis with alterations in myocardial structure (49–51). Insulin resistance is now considered to be a cardiometabolic disorder predisposing people to both CVD and diabetes. Metabolic risk factors in patients with insulin resistance are atherogenic dyslipidemia, hypertension, glucose intolerance, and a prothrombotic state (52). Further, McGavock et al., by using proton magnetic resonance spectroscopy, have shown that triglyceride accumulation occurs in human myocardium in association with diabetes mellitus and insulin resistance, much earlier than the symptoms of heart failure develop (53). Thus, the accumulation of fat and abundance of FAs is associated with an impaired cardiac efficiency and lipotoxicity.

### Hypertension

Elevated blood pressure exerted on the vessel wall is commonly referred to as hypertension. Hypertension is a well-known risk factor for dilated hypertrophy, heart failure, and stroke. About 50% of ischemic strokes are caused by hypertension with a high-risk factor for hemorrhagic stroke. The risk ratio of MI doubles when the diastolic pressure is 94 mm Hg and systolic pressure is 140 mm Hg (54). Untreated hypertension is an add-on factor for DCM, as it leads to the rapid advancement of mild subclinical DCM to the clinically visible diastolic dysfunction and then later systolic dysfunction (55). Studies have shown that the hypertensive-diabetic rats have greatest relative cardiac hypertrophy and increased interstitial fibrosis compared with only diabetic or hypertensive animals. The combination of hypertension and diabetes mellitus led to myocardial degeneration similar as observed in human patients (56). Increased blood pressure is linked with sodium intake, hyperlipidemia, insulin resistance, impaired glucose tolerance, obesity, pre-diabetes, and diabetes and can lead to pulmonary edema and heart attack. The progression of DCM leads to activation of the renin-angiotensin system, which leads to an increase in oxidative damage with cell apoptosis and necrosis in the heart thereby increasing interstitial fibrosis (57). The potent vasoconstrictor endothelin-1 (ET-1) isolated from endothelial cells has inotropic, chemotactic, and mitogenic properties, activating the renin-angiotensin-aldosterone system to increase blood pressure. Many *in vitro* studies have highlighted that high glucose activates ET-1, which mediates cardiomyocyte hypertrophy *via* mitogen-activated protein kinase (MAPK) activation (58). Genetic deletion or pharmacological inhibition of ET-1 results in salt-sensitive hypertension (59). Depending upon its presence in either medulla or cortex, the renal ET-1 has different effects on blood pressure. The upregulation of cortical ET-1 expression has been well demonstrated in various hypertensive models *via* an increase in renal vascular resistance and a reduction in glomerular filtration rate (60, 61). Further, activation of the glomerular ETA receptor leads to hypertension by enhancing production of monocyte chemoattractant protein-1 and other pro-inflammatory factors, sequestering macrophages and lymphocytes, thereby increasing sodium reabsorption (62). Clinically, it has been observed that mortality of patients suffering from MI is greater in hypertensive patients, as showed in the PROCAM study (63). Hypertension, when it coexists with diabetes, doubles the risk of cardiac failure. Other trials, 4S, CARE, and LIPID, which included patients with MI and angina pectoris, showed a 23–45% incidence of hypertension (64, 65, 166). Recent studies suggest the involvement of 12/15-LOX in the murine models of experimental hypertension by altering macrophage functions (66). Patients with essential hypertension were observed to have higher levels of 12-HETE and 12/15-LOX protein compared with control subjects (67). A randomized, double-blinded, and controlled clinical trial with patients having peripheral arterial disease (75% hypertensive) showed that patients who consumed a diet including 30 g of milled flaxseed (*n*-3-fatty acid-α-linolenic acid) for 6 months displayed significant reductions in systolic (−10 mm Hg) and diastolic (−7 mm Hg) blood pressure (68). The plasma of FlaxPAD (Flaxseed for Peripheral Arterial Disease) patients exhibited significant decreases in 5,6-, 8,9-, 11,12-, and 14,15-dihydroxyeicosatrienoic acid and 9,10- and 12,13-dihydroxyoctadecenoic acid versus controls. The study showed that inhibition of soluble epoxide hydrolase contributed to the antihypertensive effects and could be one of the pharmacological targets (68).

## Biochemical Stressors for the Development of DCM

This review further discusses biochemical mechanisms such inflammation, 12/15-LOX signaling, FA oxidation, and lipotoxicity that are directed toward increasing our understanding of novel ways for the prevention and treatment of cardiomyopathy.

### LOX Signaling and Inflammation

Inflammation is an essential biological process to restore normal tissue homeostasis after injury. The state of inflammation is mediated by upregulation of multiple signaling pathways, such as NF-κB, c-Jun NH2-terminal kinase, or p38-MAPK associated with insulin resistance, which have profound roles in diabetic complications (69, 70). Thus, overactive inflammation is unifying component of many chronic diseases eventually leading to heart failure. Since in early 1950s, several reports validates that chronic inflammation is key hallmark signature in congestive heart failure pathology. Levine et al., in 1990, documented a positive correlation between TNF-α and chronic heart failure (71). Direct correlation between many chemokines/cytokines and heart failure suggests large oxidative products, which are key regulators of the inflammatory process, exerting both pro- and anti-inflammatory effects (72, 73). The lipid-metabolizing enzymes, including different members of the LOX family, are major players in the pathogenesis of heart failure. LOXs are enzymes that metabolize polyunsaturated FAs (74). The differential prostaglandins are produced by the cyclooxygenase pathway and leukotrienes through the LOX pathway, with availability of arachidonic acid substrate leading to overactive inflammation during the healing process in heart failure pathology. Arachidonic acid serves as the substrate for LOX pathway that facilitates interaction with 5-, 12-, and 15-LOX. Within the family of LOXs, 12- and 15-LOX (referred as together 12/15-LOX) forms a subgroup of phylogenetically closely related enzymes that are highly, but not exclusively, expressed in distinct monocyte-derived cells (74). 12/15-LOX is often referred to as “leukocyte-type” 12-LOX, and has orthologs in other species such as human 15-LOX and rabbit 15-LOX-1 (75). Murine and human 12/15-LOX differ in their enzymatic activity and in their position during arachidonic acid oxygenation resulting in the predominant generation of 12-(S)-HETE by murine 12/15-LOX and 15-HETE by human 12/15-LOX (15-LOX-1), respectively (76). The pro-inflammatory role of 12/15-LOX has been evidenced in failing hearts (44, 77). Wen and colleagues demonstrated that 12/15-LOX products, i.e., 12(S)-HpETE (12(*S*)-hydroperoxyeicosatetraenoic acid) and 12(S)-HETE, are pro-inflammatory in nature and stimulate TNF-α and IL-6 expression in macrophages as a particular effect of the 12/15-LOX products. A recent report by Suzuki et al. demonstrated that the TNF-α and collagen markers were elevated with increases in 12/15-LOX expression in the STZ-induced diabetic heart (44). By contrast, 12/15-LOX knockout mice showed suppressed levels of TNF-α and collagen markers with improved cardiac function. These authors also demonstrated that administration of a 12/15-LOX inhibitor (CDC) suppressed the TNF-α levels associated with high blood glucose levels *in vitro* (44).

### FA Oxidation

The beta-oxidation of FAs is the primary mechanism for the heart to produce energy. Under resting conditions, FAs cover more than 70% of the cardiac energy needs by meeting this demand through the tricarboxylic acid cycle and electron transport chain. The excess of FAs is stored in adipose tissue. During obesity and diabetes, the adipose tissue increases in size that overspills free FAs leading to imbalance between energy demand and supply (78). An imbalance of FA oxidation or glucose oxidation leads to changes in cardiac mitochondrial metabolic energy that leads to ventricular dysfunction and to a decrease in cardiac performance. The impaired FA oxidation or increase in FA uptake in diabetes leads to an intramyocardial lipid overload (79). Myocardial abundance triggers defects in insulin signaling and activation of peroxisome proliferator-activated receptor-α (PPAR-α)/PGC-1 (80). The changes in FA oxidation lead to lipotoxicity and ceramide accumulation in cardiac tissue. Wu et al. have demonstrated that the deletion of the gene encoding aryl hydrocarbon nuclear translocator (ARNT) in the liver/pancreas leads to a diabetic phenotype with twofold increase in FA oxidation. The deletion of ARNT leads to an increase in the expression of PPAR-α and its target genes (81). FA oxidation is a major contributor of carbon substrates to ATP generation in the adult heart. However, the heart has a unique ability of metabolic flexibility and utilizes glucose, lactate, ketones, and amino acids (82). The heart has the capacity to selectively use substrate based on availability and pathophysiological state. It is well documented that glucose and lactate are preferred by the fetal heart; however, lipids are the predominant fuel in the adult heart. Animal models of cardiac hypertrophy are observed to switch their substrate metabolism, recapitulating the “fetal metabolic profile” utilizing carbohydrates as primary energy sources (83, 84). Cardiac hypertrophy models show the consistent appearance of fetal gene expression, which is considered prime trigger in the pathological remodeling of the heart.

### Lipotoxicity

Altered FA utilization and intramyocardial lipid accumulation are key major factors in the pathogenesis of DCM. The metabolic preference of the diabetic heart toward glucose leads to an increase in FAs, which leads to lipid accumulation resulting in lipotoxicity. Clinical studies suggest that congenital lipodystrophy, a rare disease causing accumulation of lipids in non-adipose tissue, is one of the causes of premature cardiomyopathy (85). In diabetic and obese animal models, accumulation of triglycerides in cardiomyocytes is often associated with impaired contractile function (86). Further, rat models of obesity have shown that defects in the leptin receptor result in an excess of fat overload in non-adipose tissues resulting in lipotoxicity (87). A study in obese Zucker rats have shown that PPAR-α-regulated genes play a crucial role in FA oxidation but become impaired in the failing heart, suggesting that metabolic dysregulation due to triglyceride overload and gene expression alteration leads to contractile dysfunction. It has also been reported that intake of unsaturated FAs increases low-density lipoprotein (LDL), triacylglycerols, and Lp(a) lipoprotein, and decreases high-density lipoprotein with a reduction in LDL cholesterol particle size, which leads to alteration in serum lipid profiles, thereby doubling the risk of CVD (88). Further, the consumption of FAs increases inflammation altering the prostaglandin balance. This impairment in the activity of desaturase (the enzyme converting linoleic acid to arachidonic acid and other n-6 polyunsaturated FAs) confers a high-risk of CVD (89–91).

### Glucotoxicity

Dysregulation of myocardium FAs use for energy source results into glucotoxicity in hyperglycemia setting developing progressive myocardial fibrosis due to glycosylation, cross-linking, and accumulation of extracellular matrix proteins (92). There is a reduction in glucose transporter expression, which inhibits glucose translocation from plasma membrane to the cell in DCM. During ischemia, PPAR’s inhibits insulin’s action that reduces the rates of glycolysis and pyruvate oxidation resulting in shutdown of glucose metabolism to the hexosamine biosynthesis pathway (93–95). The inappropriate hexosamine biosynthesis metabolism leads to the production of reactive oxygen species (ROS) and the increased formation of intracellular advanced glycosylation end-products (AGEs). AGEs and hexosamine biosynthesis impacts the sarco-endoplasmic reticulum Ca^2+^-ATPase and the Ca^2+^ release channel, ryanodine receptor 2, leading to abnormal cardiac relaxation and contractility (96, 97). Wang et al. showed a novel role of active heparanase in modulating cardiac metabolism *via* cross talk between endothelial cell and cardiomyocyte to increase lipoprotein lipase secretion after hyperglycemia (98). The study showed that high glucose is a common stimulus for latent heparanase secretion from the endothelial cells and promotes its uptake into the cardiomyocyte (99). Presence of latent form of heparanase in the cardiomyocyte leads to the significant shift in the expression of apoptosis-targeted genes, providing an acute cardioprotective effect indicating diversified roles of heparanase in DCM and heart failure pathology (99).

## Gender Specificity, Circadian Rhythm, and Aging Modulators in DCM

Although gender-specific studies have shown that females tend to develop cardiac complications 10–15 years later than men (100), it has also been proven that women with diabetes and hypertension have a greater risk of developing CVD (101, 102). Clinical reports have also shown that if CVD presents at a younger age in women, it is more detrimental (103). The female heart tolerates stress, such as ischemic insults, better than the male heart. In diabetes, the estrogen in females may interact with certain risk factors, which may be deleterious to overall cardiac function. A study published by Peters and colleagues showed a sex-specific risk of stroke conferred by diabetes. Meta-analysis conducted for 64 cohorts including 775,385 individuals who did not show any signs of cardiac problems at baseline determined that 12,539 individuals experienced fatal or non-fatal stroke events (104). The circadian clock allows the body to adjust metabolic cycles according to the shift in day and night. These circadian oscillations impact physiological parameters of a cardiovascular function, thermoregulation, lipid, and glucose metabolism. Studies have shown that circadian misalignment accelerates diabetes due to disruption of glucose-stimulated insulin secretion and beta-cell loss (105). Two transcription factors, CLOCK and BMAL1, transcriptionally control the circadian clock by binding to E-boxes of target genes upon heterodimerization (106, 107). In the heart, BMAL1 regulates substrate utilization, FA and glucose metabolism, and the PI3K/AKT/GSK3β signaling axis. The cardiomyocyte-specific knockout of BMAL1 leads to age-onset cardiomyopathy, reducing lifespan with impaired FA and glucose metabolism (108). Our group has shown that genetic disruption of BMAL1 results in diastolic dysfunction exacerbating extracellular matrix remodeling, with the increase in expression of a pro-inflammatory gene profile signifying early cardiac aging in mice (109). Aging, diabetes, and hypertension have similar effects on heart dysfunction, resulting in LV hypertrophy and stiffness. Aging leads to an increase in cardiovascular stiffness contributing to the development of fibrosis. Fibrosis increases collagen cross-linking due to the formation of AGEs (110). Rodent studies have shown that at 16 weeks of age, structural and functional changes are observed in both the heart and kidney, in the Zucker diabetic fatty model of type 2 diabetes. Aging is widely impacted by the diverse intake of dietary ingredients (111). Both insulin resistance and hyperglycemia can occur with an increase in n-6 FAs, which further magnifies the inflammatory response in heart failure in aging (112). Clinically, with advancing age, the risk to people with the normal systolic function of having heart failure is 50%. However, diastolic dysfunction occurrence is higher in women with hypertensive heart disease and diabetes.

## Emerging Molecular Pathways for DCM

**Figure 2:**
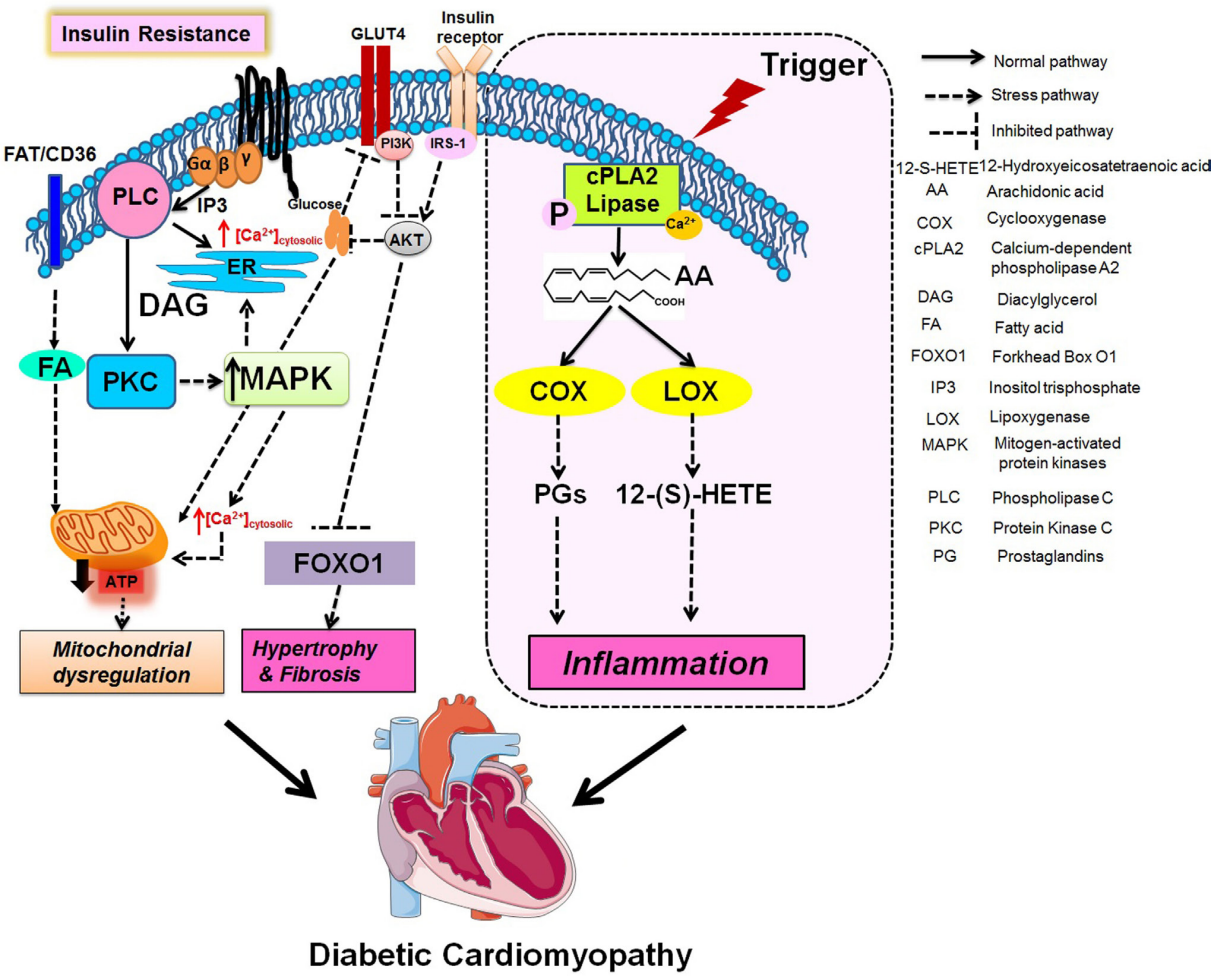
**Integration of metabolic and fatty acids (FAs) metabolizing enzymes pathway in mitochondrial dysregulation, fibrotic hypertrophy, and chronic inflammation**.

Diabetic cardiomyopathy leads to structural and functional changes in myocardium by activation of signaling pathways, i.e., altered calcium signaling, increased ROS, ceramides, hexosamines, advanced glycation end-products, inflammatory signaling, and changes in many transcriptional regulators along with 12/15-LOX, which contribute toward the pathogenesis as shown in Figure 2 (113). Being a multifactorial disease, the crosstalk between immune cell dysregulation, cardiomyocytes, endothelial cells, and fibroblasts leads to the impairment of several signaling pathways leading to the etiology of DCM discussed above. The MAPK family is essential for cardiac survival, and MAPK signaling impairment has been well studied in diabetic tissue (114, 115). STZ-induced diabetic rats have shown upregulated p-38-MAPK activity (116). The diabetic myocardium mediates apoptosis *via* ASK1, a MAPKKK signaling molecule (117). FoxO (forkhead box-containing protein, O subfamily-3, -4) proteins are major targets for the maintenance of cardiac function and stress responsiveness by regulating cardiac growth, insulin signaling, and glucose metabolism in the heart (118–120). Also, several pathways including mammalian target of rapamycin, microRNAs, Pim-1 (proviral integration site for moloney murine leukemia virus-1), endoplasmic reticulum stress, and unfolded protein responses are dysregulated in DCM (121).

## Development of Novel Targets for DCM

Being a multifactorial disease; currently, there is no specific therapy for DCM. Table 3 describes established rodent models to study DCM for novel treatment. Since insulin resistance is one of the leading causes of the pathogenesis of cardiomyopathy, insulin signaling is one of the targets. Anti-diabetic drugs, such as metformin, act on the metabolic target AMPK and may confer cardiovascular benefit (122, 123). Similarly, maintaining a proper diet and exercise can reduce the risk of diabetes and CVDs (124). Compounds modulating free FA metabolisms, such as perhexiline, trimetazidine, ranolazine, and amiodarone, have been shown to reduce lipotoxicity (125). The resveratrol-activated NAD-dependent protein deacetylase Sirt1 is a potent target as it plays a role in lowering blood glucose and increasing insulin sensitivity (126). Cardiac excitation–contraction coupling and insulin signaling dysregulation can also be improved by cell-based and genetic ablation therapy, which are among the robust strategies for treating CVDs (127). With the emergence of potent pro-inflammatory and anti-inflammatory roles of arachidonic acid metabolites such as 20-HETE, 12-HETE, and soluble epoxide hydrolase in diabetes and CVD (77, 128, 129), lipid mediators and their enzymes are among the potent future therapeutic targets to protect against the initiation and progression of DCM. The underlying concept “targeted or aggressive metabolomic” approach used by West et al. to the targeted metabolic profiling of cardiac tissue in dilative cardiomyopathy can be one the promising tool to delineate the DCM in future (130). With the rapid evolvement of the targeted metabolomics that aims to measure the endogenous metabolites in a cell or body fluid providing the functional readout. All the changes in the functional reads outs, such as shifts in the homeostasis of key lipids, carbohydrates, or amino acids, are associated with genetic variants. The first genome-wide association study with metabolomics (KORA study) using the quantitative measurement of 363 metabolites in serum of 284 male participants (131). The study found an association of single nucleotide polymorphisms with considerable differences in the metabolic homeostasis. The study found four genetic variants in genes coding for enzymes (FADS1, LIPC, SCAD, and MCAD) where the corresponding metabolic phenotype (metabolite) clearly matches the biochemical pathways in which these enzymes are active. The study clearly links genetic polymorphisms induce changes in the metabolic make-up of the human population opening a whole new field for personalized health care based on a combination of genotyping and metabolic characterization. The adaptation of sedentary lifestyle and eating habits has led to the increase in DCM about 5% of the global population (132). A blinded randomized personally tailored dietary intervention with consistent alterations to gut microbiota resulted in significantly lower postprandial responses (133). Accurate personal dietary recommendation using personal and microbiome features will be valuable in lowering the risk of DCM and associated inflammatory, metabolic, and neoplastic multifactorial disorders and can be effective clinical decision-making scheme.

**Table 3:** **Established rodent diabetic cardiomyopathy models for the development of novel therapy**.

S. No	Model	Metabolic, biochemical, and functional outcomes	Reference
1	Streptozotocin	Lipotoxicity, hyperglycemia, diastolic and systolic dysfunction, hypertrophy, inflammation, fibrosis, glucose and fatty acid (FA) oxidation, mitochondrial dysfunction, oxidative stress, Ca^2+^ impairment	(32, 33, 35, 134–138)

2	Beta-cell overexpression of calmodulin	Reduced insulin levels, hyperglycemia and hyperlipidemia, impaired cardiac function, hypertrophy, inflammation apoptosis, FA oxidation, mitochondrial dysfunction, Ca^2+^ impairment	(98, 139–144)

3	Non-obese diabetic	Autoimmune diabetes, hyperglycemia, high cholesterol, diastolic and systolic dysfunction, steatosis, immune dysregulation	(145–151)

4	Leptin-deficient obese mice	Leptin deficiency, appetite suppression hyperglycemia and hyperlipidemia, diastolic dysfunction, hypertrophy, inflammation steatosis, apoptosis, decreased glucose oxidation and increased FA oxidation, mitochondrial dysfunction, Ca^2+^ impairment	(15, 41, 93, 135, 152–159)

5	Type 2 diabetic Lepr deficient	Hyperglycemia and hyperlipidemia, diastolic and systolic dysfunction, hypertrophy, inflammation steatosis, apoptosis, decreased glucose oxidation and increased FA oxidation, mitochondrial dysfunction, oxidative stress, Ca^2+^ impairment	(13, 41, 147, 155, 158, 160–165)

## Future Perspectives

Application of novel imaging technology and the use of biochemical markers (protein, enzymes, and metabolites) in clinical and pre-clinical models indicate that metabolic and biochemical stressors promote heart dysfunction in the diabetes setting. With the advancement of current research programs in pre-clinical and clinical models, it is possible to determine whether the DCM is of a dilated, hypertrophy, non-compaction or restrictive type, or of a combination of these. Whether the distinct target is at the enzymatic (LOX), mitochondria centric, ion-channel related, or defective chemokine-cytokine signaling level. Thus, additional studies with the major emphasis on metabolic and biochemical stressors in the advancement of heart function and dysfunction will determine future treatment(s). Despite the high mortality in diabetes patients due to heart failure, a number of questions regarding what triggers DCM or amplifies progressive cardiomyopathy remain unclear; therefore, urgent research on novel therapies is needed to meet the demand of personalized and precise medicine in the twenty-first century (165).

## Author Contributions

GH conceptualized the outline, edited the review, and approved for submission. VK prepared the first draft and edited the input from GH.

## Conflict of Interest Statement

The authors declare that the research was conducted in the absence of any commercial or financial relationships that could be construed as a potential conflict of interest.
